# The qualitative and quantitative characteristics of serous endometrial carcinoma on MRI: applying a novel nomogram for predicting an aggressive histological type

**DOI:** 10.3389/fonc.2025.1472250

**Published:** 2025-03-14

**Authors:** Rennan Ling, Hongtao Jin, He Zhang

**Affiliations:** ^1^ Department of Radiology, Shenzhen People's Hospital, 2nd Clinical Medical College of Jinan University, 1st Affiliated Hospital of Southern University of Science and Technology, Shenzhen, China; ^2^ Department of Pathology, Shenzhen People's Hospital, 2nd Clinical Medical College of Jinan University, 1st Affiliated Hospital of Southern University of Science and Technology, Shenzhen, China; ^3^ Department of Radiology, Obstetrics and Gynecology Hospital, Fudan University, Shanghai, China

**Keywords:** serous carcinoma, endometrial cancer, magnetic resonance imaging, quantitative, nomograms

## Abstract

**Objectives:**

To comprehensively describe MRI characteristics of serous endometrial carcinoma (SEC) and distinguish SEC from endometrioid endometrial carcinoma (EEC).

**Methods:**

We retrospectively recruited 62 patients from a tertiary center with pathologically proven endometrioid cancers (37 SEC and 25 EEC) as the training set. MRI image interpretation was blindly interpreted by two experienced radiologists with consensus reading. Both qualitative and quantitative characteristics on MRI were recorded case by case. Histological findings were retrieved from the hospital information system. Fifty-four samples (27 SEC and 27 EEC) from the external hospital were treated as the testing set.

**Results:**

The qualitative MRI characteristics had no statistical difference between the SEC and EEC groups in the training set. SEC more often invaded the deep myometrium than EEC (*p* = 0.03). The signal intensity (SI)_T2_Ratio, SI_contrast_Ratio, Lesion_area_Ratio, and Volume_area_Ratio in the SEC group were 1.35 ± 0.36, 0.77 ± 0.18, 0.25 ± 0.24, and 0.22 ± 0.26, respectively. The SI_T2_Ratio, SI_contrast_Ratio, and Volume_area_Ratio showed statistically significant differences between SEC and EEC (*p* < 0.05). The highest discriminative index for distinguishing SEC from EEC was SI_contrast_Ratio with an area under the curve (AUC) of 0.7533 (95% CI: 0.627–0.878). A predictive nomogram achieved an AUC of 0.814 (95% CI: 0.614–0.968), a sensitivity of 1.0, and a specificity of 0.60 in the testing set.

**Conclusions:**

This study developed and validated a nomogram model to predict SEC patients based on clinical and quantitative MRI features, which can be used in distinguishing SEC from EEC.

## Introduction

Endometrial cancer (EC) is the predominant gynecological malignancy globally. As per the fifth edition of the World Health Organization (WHO) Classification of Tumors, Female Genital Tumors, EC encompasses a wide spectrum of histological types, among which endometrioid endometrial cancer (EEC) is the most frequent histologic type, typically associated with a good prognosis. In contrast, serous endometrial carcinoma (SEC) constitutes roughly 10% of cases but is responsible for up to 40% of EC-related fatalities due to its aggression, due to chemotherapy resistance and earlier relapse (1). Pathologically distinguishing high-grade EEC from SEC is challenging, as both exhibit TP53 gene mutations (2, 3). Specifically, histologic types and lymphovascular invasion (LVSI) are the important aggressive factors affecting the prognosis. With the growing recognition of the importance of determining prognosis and treatment planning, histologic types and LVSI are included in the latest 2023 International Federation of Gynecology and Obstetrics (FIGO) staging for endometrial cancer (4). Therefore, the accurate preoperative prediction of aggression is increasingly essential and crucial. Magnetic resonance imaging (MRI) plays a crucial role in diagnosis, preoperative staging, posttreatment assessment, and follow-up (5–9). Notably, detailed accounts of EC on MRI remain scant (6). Precise preoperative determination of histological types is of paramount interest to gynecologists for informed clinical decision-making (10). Hence, this study aimed to fulfil two objectives: firstly, to offer a comprehensive depiction of both the qualitative and quantitative characteristics of EEC and SEC on MRI from two tertiary institutions; secondly, to delineate the MRI characteristics distinguishing SEC from EEC, assess their congruence with pathological findings, and develop a nomogram predictive model specifically for SEC patients.

## Materials and methods

### Study population

**Figure 1:**
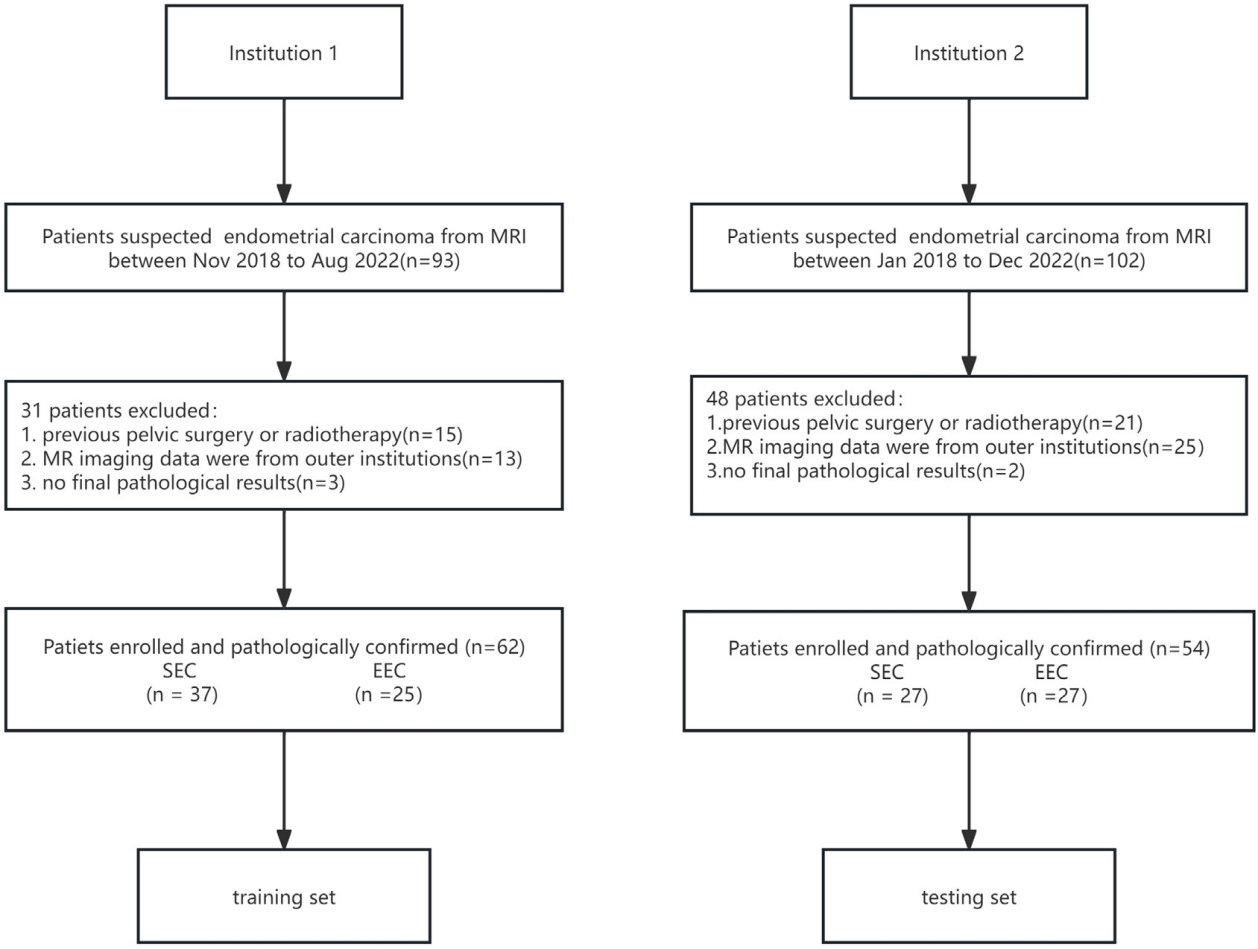
The flowchart of patient selection.

The institutional review board approved this retrospective study, and the requirement for informed consent was waived for all participants. The inclusion criteria were as follows: 1) performing MRI within preoperative 2 weeks, 2) maximal tumor diameter larger than 1 cm on MR imaging, 3) not having non-gynecological malignancy at the time of their participation in this study, 4) no chemo-radiotherapy and/or immunosuppressive therapy before MRI, 5) complete clinical data, 6) complete MRI protocols and no image quality pitfalls, 7) clinical FIGO stage I~III, and 8) being able to follow up. From November 2013 to August 2018, 62 patients with pathologically confirmed SEC (*N* = 37, mean age: 62.0 ± 7.2 years old) and EEC (*N* = 25, mean age: 53.5 ± 8.99 years old) were retrieved from the institutional picture archiving and communication system (PACS) from institution 1. A total of 54 patients (mean age: 55.3 ± 10.1 years old), consisting of 27 SEC samples and 27 EEC samples, from institution 2 between January 2018 and December 2022 were included as an external, independent test datasheet (**Figure 1**). All included patients were pathologically confirmed by invasive surgery (laparoscopy or laparotomy) from two tertiary centers. FIGO staging, pathological types, immunohistochemical staining results, and laboratory examinations were collected through the hospital information system.

### MRI protocol and MRI characteristics

In institution 1, MRI was performed using a 1.5-T MR scanner (Magnetom Avanto, Siemens, Erlangen, Germany) with a phased-array eight-channel sensitivity encoding abdominal coil. Routine MRI protocols used for the assessment of pelvic masses included axial turbo spin-echo (TSE) T1-weighted imaging (T1WI), sagittal TSE T2-weighted imaging (T2WI), and axial/sagittal TSE fat-suppressed T2WI (fs-T2WI). Diffusion-weighted imaging (DWI) using a two-dimensional sequence of echo-planar imaging (EPI) was performed in the axial plane with a parallel acquisition technique by using *b* = 0, 100, and 800 s/mm^2^. Pelvic-enhanced imaging was acquired at multiple enhancement phases in sagittal and axial planes. Dynamic contrast-enhanced T1WI gradient-echo images were obtained at 30, 60, and 90 s in the axial plane and 120 s in the sagittal plane after injection of gadobutrol (Magnevist; Bayer Schering, Berlin, Germany) at an injection flow of 2 mL/s and a dose of 1 mL/kg body weight.

In institution 2, all scans were performed on a 3.0-T MRI scanner (Siemens Magnetom Skyra, Erlangen, Germany) with a phased-array eight-channel sensitivity encoding abdominal coil. The conventional MRI protocols are the same as institution 1. DWI was performed using *b* = 0, 500, and 1,000 s/mm^2^. Contrast-enhanced fat-suppressed T1WI volumetric interpolated breath-hold examination was performed at 60 s after injection of gadobutrol (gadopentetate dimeglumine injection; Beijing Beilu Pharmaceutical Co., Ltd., Beijing, China) at a dose of 1 mL/kg body weight. The detailed basic scanning parameters are summarized in the supplementary table.

**Figure 2:**
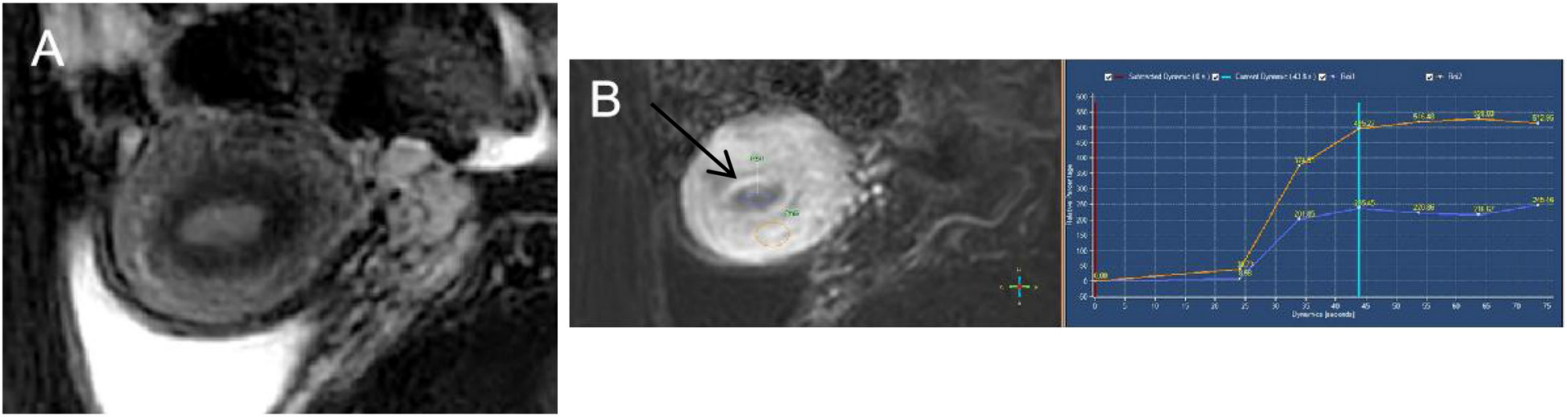
Endometrioid endometrial carcinoma in a 46-year-old woman. **(A)** On sagittal T2WI, the tumor has moderate signal intensity and is found in the uterine cavity. **(B)** The focal myometrium protrudes toward the lesion (white arrow). The time–intensity curve was measured with a region of interest (ROI) placed on both endometrium (ROI1) and cancer tissues (ROI2).

All MRI characteristics were evaluated by two experienced radiologists (both with more than 10 years of experience in gynecology MR knowledge) on a PACS terminal server referring to a reading consensus. On T1WI, the hypo-, iso-, and hyperintensities were similar for the pelvic fluid, pelvic wall muscle, and subcutaneous fat signal; on T2WI, the hypo-, iso-, and hyperintensities were similar to the pelvic bone, pelvic wall muscle, and endometrium signal; on high *b*-value DWI images, the low-, intermediate-, and high-signal intensities were similar to the pelvic fluid, myometrium, and endometrium. Apparent diffusion coefficient (ADC) values were manually measured on a commercially available workstation on ADC map images. Signal intensity T2 ratio (SI_T2_Ratio) means that the lesion’s signal intensity is divided by the uterus’s signal intensity on T2WI; contrast signal intensity ratio (SI_contrast_Ratio) means that the lesion’s signal intensity is divided by the uterus’s intensity on the latest phase of contrast images which is approximately 60 s after contrast injection (**Figure 2**); lesion area ratio (Lesion_area_Ratio) means that the largest lesion’s area is divided by the uterus’s area on sagittal T2WI; and lesion volume ratio (Volume_area_Ratio) means that the volume of a lesion is divided by the uterus’s volume on sagittal T2WI. All lesions were calculated and measured using the ITK-Snap software by one experienced radiologist and another radiologist will review the results one by one. Once an agreement is reached, the final ROI is defined and documented. If there is a disagreement, negotiation is used to achieve consensus. This collaborative effort helps ensure that the ROI is as accurate and reliable as possible, reducing the likelihood of errors due to individual bias or oversight.

The MRI images were assessed, including 1) the lesion maximum diameter and size, 2) the signal intensity and the ratio of signal intensity, 3) myometrial invasion (less or deep myometrial invasion), and 4) the presence of node enlargement and coexistent etiologies.

### Clinical and pathologic features

The clinical characteristics included the onset age, body mass index (BMI), the serum carbohydrate antigen 125 level (CA125), and the serum human epididymal protein 4 level (HE4). The pathological characteristics included pathological types, lymphatic metastatic node (LNM), LVSI, and Ki-67 expression. All patients were clinically staged according to the 2009 FIGO staging system.

### Statistics

All analyses were performed using SPSS (version 23.0, IBM, Armonk, NY, USA: IBM Corp). Continuous variables were reported as medians and ranges if the data were not normally distributed and were compared with the Kruskal−Wallis test. Normally distributed variables were presented as the mean and standard deviation and were compared with the Student’s *t*-test. Categorical variables were reported as percentages and compared with the Mann−Whitney *U* test. The consistency of inter-/intraoperator measurements was also evaluated by the intraclass coefficient test. A value of *p <*0.05 was considered statistically significant. A predictive nomogram was constructed based on multivariable logistic analysis to quantitatively identify SEC and was evaluated by ROC and decision curve analysis (DCA) (11).

## Results

### Patient characteristics

**Figure 3:**
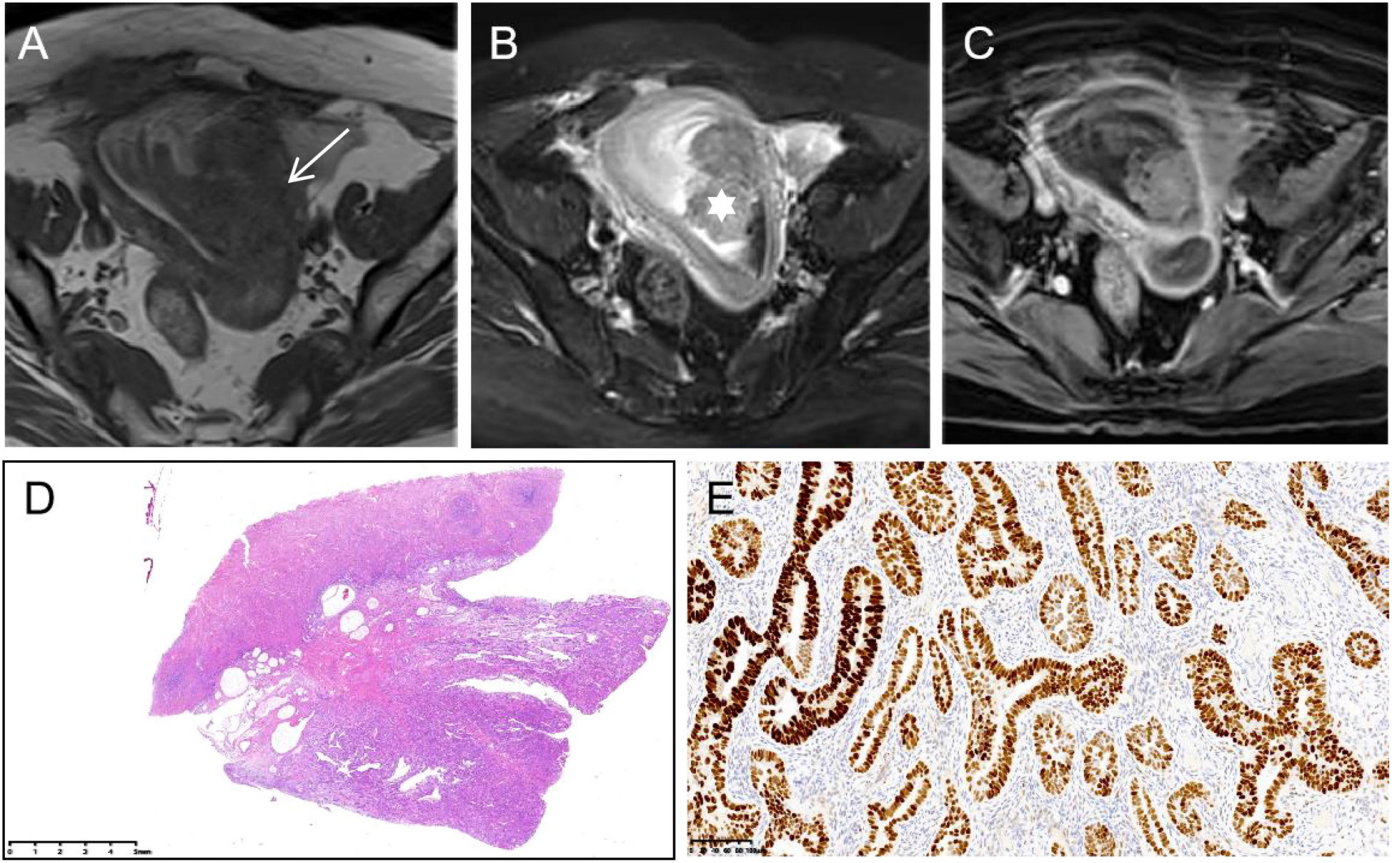
Serous endometrial carcinoma in a 57-year-old woman. **(A)** On axial T1WI, tumor tissues (white arrow) show isointensity. **(B)** On axial T2WI with fat saturation, the tumor shows hyperintensity, mainly inside the endometrium (white star). **(C)** Axial contrast-enhanced T1WI with fat saturation presents obvious enhancement. **(D)** The gross species shows polypoid growth (HE stain ×0.5) with strong positivity for p53 (**(E)**, ×0.5).

**Figure 4:**
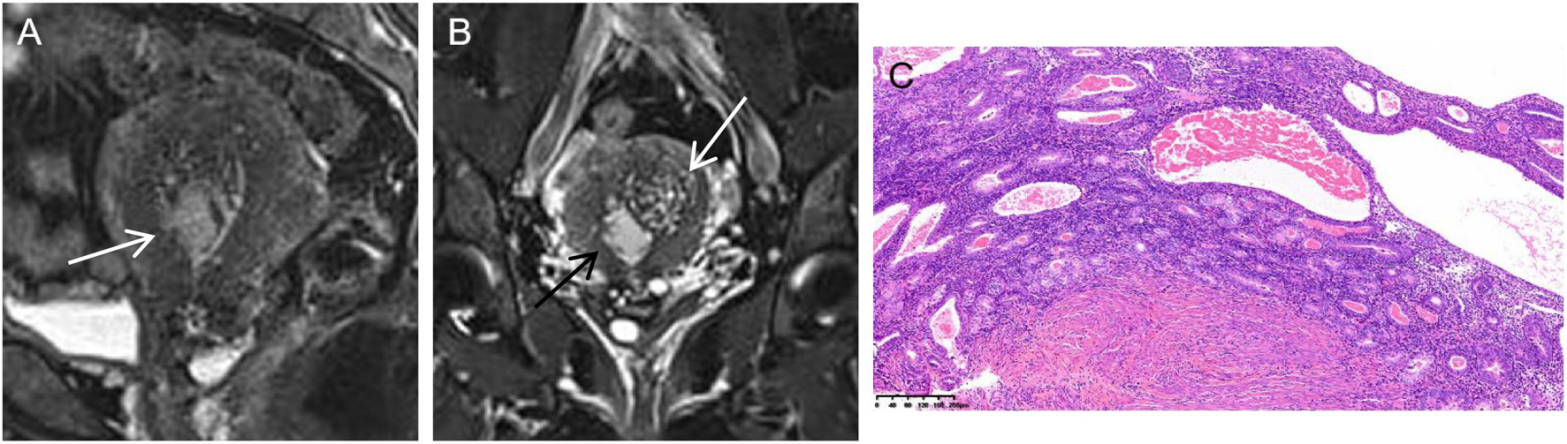
Serous endometrial carcinoma in a 46-year-old woman. **(A)** On sagittal T2WI, there was an unclear boundary between the cancer tissues and junctional zone (white arrow). **(B)** On coronal T2WI with fat saturation, the tumor mass (black arrow) originated from adenomyosis (white arrow). **(C)** Histology disclosed that both the adenomyosis and muscular tissues were involved by the cancer tissues (HE stain ×10).

In this study, the onset age of the SEC group (62.0 ± 7.19 years) was higher than that of the EEC group (53.5 ± 8.99 years). There was a statistically significant difference (*p* > 0.05). The serum CA125 level was higher in the SEC group than in the EEC group, and the difference was statistically significant (123.1 ± 329.6 U/mL vs. 27.2 ± 25.9 U/mL, *p* = 0.014) (**Figure 3**). The serum HE4 level was higher in the EEC group than in the SEC group with no statistically significant difference (82.4 ± 41.2 pmol/L vs. 167.9 ± 210.6 pmol/L, *p* = 0.342) (**Figure 4**). The Ki-67 level in the SEC group was higher than that in the EEC group with no statistically significant difference (42.9% ± 19.8% vs. 39.0% ± 22.3%, *p* = 0.557). All EEC cases were low risk (grades 1 and 2) and all SEC cases were high risk (grade 3). The lesions of the SEC group more often invaded the deep myometrium compared to those of the EEC group (17/37, 45.9% vs. 3/22, 12%, *p* = 0.011), which represented more aggressive biological abilities. The cases with LNM and LVSI both in the SEC and EEC groups had no statistically significant differences compared to the non-LNM and non-LVSI cases (*p* > 0.05). In both groups, the most common coexistent etiology was myoma (six cases in the EEC group and seven cases in the SEC group). The five cases with co-occurrent endometrial polyps or hyperplasia were observed only in the SEC group. The basic characteristics of the included samples are summarized in **Table 1**.

**Table 1:** The baseline characteristics of institution 1.

Characteristic	SEC (*N* = 37)	EEC (*N* = 25)	*p*-value
**Age (average ± SD)**	62.0 ± 7.19	53.5 ± 8.99	0.08*
**BMI**	24.9 ± 3.65	25.7 ± 3.24	0.725
**CA125 (U/ml)**	123.1 ± 329.6	27.2 ± 25.9	0.014*
**HE4 (pmol/L)**	82.4 ± 41.2	167.9 ± 210.6	0.342
**Ki-67 (%)**	42.9 ± 19.8	39.0 ± 22.3	0.557
Grade			
Low grade (grades 1–2)	0 (0%)	25 (100%)	
High grade (grade 3)	37 (100%)	0 (0%)	–
Myometrial invasion			
≥1/2	17 (45.9%)	3 (12%)	
<1/2	20 (54.1%)	22 (88%)	0.011*
Lymph node			
Regional lymph node involvement	8 (21.6%)	3 (12%)	
No regional lymph node involvement	29 (78.4%)	22 (88%)	0.335
Lymphovascular invasion			
Yes	14 (37.8%)	6 (24%)	
No	23 (62.1%)	19 (76%)	0.257
**Complications**	15 (40.5%)	7 (28%)	0.491
Endometrial polyp and/or hyperplasia	5 (13.5%)	0 (0%)	
Myoma, adenomyosis, ovarian cancer, etc.	10 (27%)	7 (28%)	

*p < 0.05.

The bold text is the classification lable.

### Qualitative and quantitative MRI characteristics

Briefly, most lesions displayed medium- to high-signal intensity on both T2WI (SEC: 34/37, 91.8% and EEC: 25/25, 100%) and DWI (SEC: 37/37, 100% and EEC: 25/25, 100%). The SEC group had an average ADC value of 0.899 ± 0.210 ×10^−3^ mm^2^/s, whereas the EEC group had an average of 0.857 ± 0.139 × 10^−3^ mm^2^/s. On contrast-enhanced T1WI, most of them showed mild enhancement at 60 s after contrast injection (SEC: 27/35, 70.3% and EEC: 22/25, 88%), which presented a rapid flush-out effect. The conventional MRI characteristics, i.e., the signal intensity of T2WI, DWI, and contrast-enhanced T1WI and the ADC value, were not statistically significantly different between the two groups (*p* > 0.05, **Table 2**).

**Table 2:** MR characteristics of institution 1.

MRI characteristics	SEC (*N* = 37)	EEC (*N* = 25)	*p*-value
Qualitative characteristics			
T2WI signal			
Hypointensity	3 (8.1%)	0 (0%)	
Isointensity	12 (32.4%)	5 (20%)	
Hyperintensity	22 (59.5%)	20 (80%)	0.122
DWI signal			
Hypointensity	0 (0%)	0 (0%)	
Isointensity	7 (18.9%)	12 (48%)	
Hyperintensity	30 (81.1%)	13 (52%)	0.078
Enhancement type			
Hypointensity	26 (70.3%)	22 (88%)	
Isointensity	7 (18.9%)	0 (0%)	
Hyperintensity	4 (10.8%)	3 (12%)	0.273
Quantitative characteristics			
**Mean ADC value (mean ± SD) × 10^−3^ mm^2^/s**	0.899 ± 0.210	0.857 ± 0.139	0.358
**SI_T2_Ratio**	1.35 ± 0.36	1.65 ± 0.63	0.026*
**SI_contrast_Ratio**	0.77 ± 0.18	0.63 ± 0.11	0.001*
**Lesion_area_Ratio**	0.25 ± 0.24	0.22 ± 0.13	0.606
**Volume_area_Ratio**	0.22 ± 0.26	0.11 ± 0.07	0.045*

*p < 0.05.

The bold text is the classification lable.

In the quantitative MRI characteristics, the values of SI_T2_Ratio, SI_contrast_Ratio, Lesion_area_Ratio, and Volume_area_Ratio in the SEC group were 1.35 ± 0.36, 0.77 ± 0.18, 0.25 ± 0.24, and 0.22 ± 0.26, respectively. The SEC group had significantly greater SI_T2_Ratio, SI_contrast_Ratio, and Volume_area_Ratio values compared to the EEC group with statistically significant differences (*p* = 0.026, 0.001, and 0.045, respectively). The highest discriminative index for distinguishing SEC cases from EEC cases was SI_contrast_Ratio (AUC: 0.7533; 95% CI: 0.627–0.878). Taking the cutoff SI_contrast_Ratio value of 0.635 for SEC identification, the sensitivity and specificity were 0.8 and 0.6, respectively. The intraclass correlation coefficients for SI_T2_Ratio, SI_contrast_Ratio, Lesion_area_Ratio, and Volume_area_Ratio were 0.912, 0.866, 0.957, and 0.791, respectively. For interoperator measurements, the consistency values were 0.924, 0.804, 0.983, and 0.851, respectively.

### Nomogram construction

**Figure 5:**
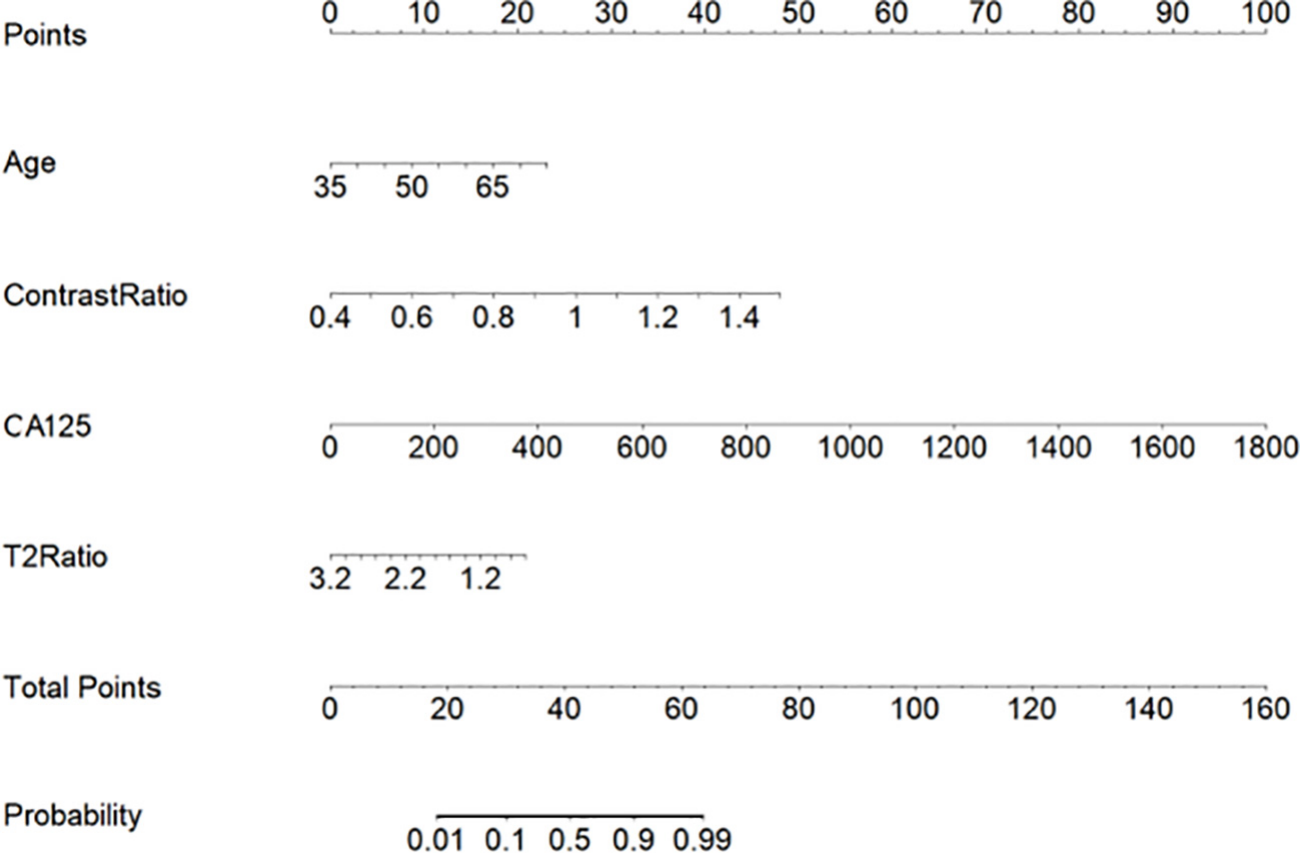
A nomogram predictive model for SEC patients.

**Figure 6:**
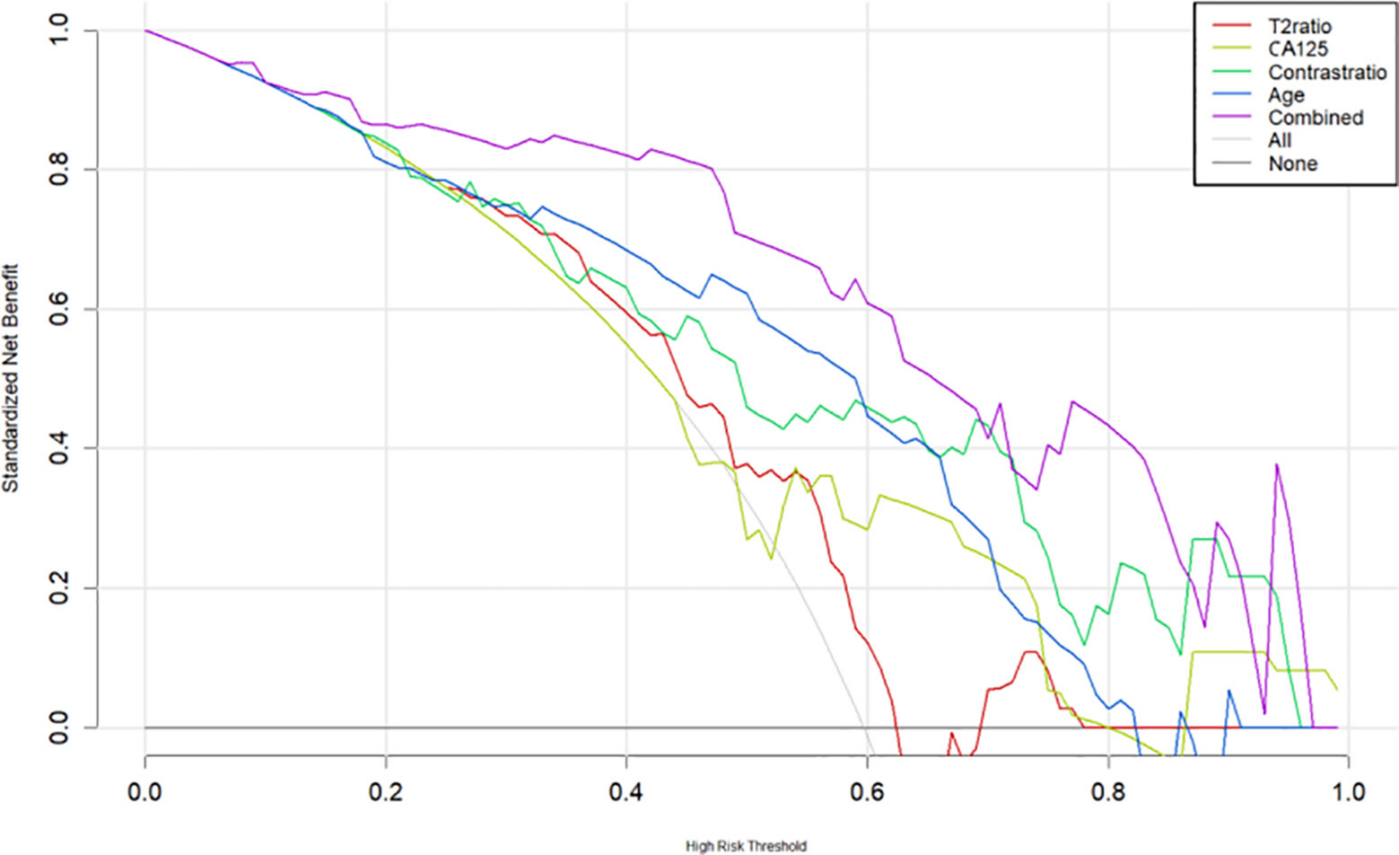
The decision curve analysis (DCA) curve for the nomogram.

The multilogistic regression test was used with the following 12 clinical and MRI features: onset age, BMI, CA125, HE4, Ki-67, LNM, LVSI, SI_T2_Ratio, SI_contrast_Ratio, Lesion_area_Ratio, Volume_area_Ratio, and ADC value. The onset age, CA125, SI_T2_Ratio, and SI_contrast_Ratio were statistically significantly different between the SEC group and the EEC group in the training set, which were selected and used to develop a nomogram predictive model for SEC patients (**Figure 5**). The nomogram achieved an AUC of 0.814 (95% CI: 0.614–0.968), a sensitivity of 1.0, a specificity of 0.60, a positive predictive value of 0.44, and a negative predictive value of 1.0 in the external testing set. The DCAs of the nomogram for the identification of SEC patients in the testing set demonstrated a net benefit when the threshold was in the range between 0.1 and 0.7 (**Figure 6**).

## Discussion

In this study, we first described the MR imaging from both the qualitative and quantitative characteristics in cases recruited from two medical centers. To prevent a potential source of bias between the two institutions, we chose the rate of signal intensity. Moreover, all MRI characteristics were evaluated by two experienced radiologists on a PACS terminal server referring to a reading consensus. We found that the qualitative MRI characteristics, such as the signal intensity of T2WI, DWI, and contrast-enhanced T1WI, did not help in differentiating SEC from EEC. The quantitative MRI characteristics, such as the values of ADC, SI_T2_Ratio, SI_contrast_Ratio, Lesion_area_Ratio, and Volume_area_Ratio, provided useful information for improving discriminative abilities. In all quantitative MRI characteristics, the value of SI_contrast_Ratio yielded the best performance, with a sensitivity of 0.8 and a specificity of 0.6. Therefore, we developed a nomogram predictive model using four clinical and MRI features for SEC patients. The computerized model combining four clinical and MRI features had a better performance than using each feature alone, which can distinguish SEC patients from EEC patients satisfactorily.

SEC is the most malignant histologic type of endometrial cancer with a relatively poor prognosis. The majority of patients affected by SEC are postmenopausal women who experience irregular bleeding. In approximately 40%–50% of cases that undergo surgical staging, there is evidence of spread beyond the uterus, most commonly to the lymph nodes, peritoneal surfaces, and the omentum (12). Histopathologically, tumors typically arise in a background of atrophic endometrium or in an endometrial polyp, exhibiting complex papillary and/or glandular architectural features in most cases (13). The cytology is high grade, characterized by significant nuclear variation in size and shape, large nuclei, and active cell division (3). Nevertheless, differentiating SEC on conventional MRI can be challenging due to similarities in appearance with other histologic types. In our study, SEC was difficult to distinguish preoperatively as it appeared on MRI either as polyps or originated from pre-existing adenomyotic tissues.

Herein, we first used both quantitative and qualitative MRI characteristics to predict SEC cases. In this study, compared to conventional qualitative MRI characteristics, quantitative characteristics provide more useful information in the prediction of SEC cases. The SI_contrast_Ratio showed the best discriminative ability compared to the other characteristics. In the SEC group, higher SI_contrast_Ratio values were recorded, reflecting a more aggressive ability. The other characteristic indices focusing on the contour features made no differences between the groups. Furthermore, we found that the model combined with clinical and MRI features could help improve the diagnostic performance compared with using each feature alone. Such a model can also be validated in an independent external dataset. Recent studies with MR radiomics showed good capability reflecting the aggressive abilities of EEC (14). By subtracting heterogenic signatures from radiologic images, radiomics studies could help discriminate the subtypes of cancers, stratify the risk of recurrence, and tailor adjuvant treatment (15–18). The number of studies is still limited in EC subtype prediction using imaging data. Our study corroborated the view that some quantitative parameters could be used as potential biomarkers to reflect the histologic types. In a recent study, the authors reported that ADC values inversely correlated with tumor cellularity in EC (19). In this study, we did not observe ADC value differences in the different histologic types of EC. In another study using conventional MRI characteristics data, the authors reported that SEC had a more heterogeneous signal intensity, which was prone to peritoneal dissemination aside from abnormal ascite accumulation compared to EEC (6). Our nomogram was based on clinical and MRI features. Our nomogram’s curve analysis and external validation show that it has good discriminant and calibration abilities. With Nomogram, we can effectively screen out SEC patients from EEC patients.

This study had several limitations. First, the number of included cases used to train the mixed-diagnostic models was relatively small, and further studies can be carried out using a larger sample. Second, although the calculation consistency of both inter- and intraoperators was good in this study, we feel that this may be biased in some less-experienced operators and may influence the final conclusion. Third, in this study, we did not outline the lesion as most radiomics studies used (20), which may eliminate some subjective bias by different users. Fourth, using different MRI scanners between two institutions may introduce a potential source of bias, and even though we used ratios for comparison, we still need to expand the sample size.

In summary, our results suggest that quantitative MRI characteristics can provide useful information for SEC and EEC patients. Furthermore, we developed a nomogram predictive model for SEC patients incorporating four clinical and MRI features. This model offers a valuable tool distinguishing SEC patients from EEC patients, potentially enhancing clinical decision-making.

## Data Availability

The original contributions presented in the study are included in the article/**Supplementary Material**. Further inquiries can be directed to the corresponding author.
