# Supplementary material for: The qualitative and quantitative characteristics of serous endometrial carcinoma on MRI: applying a novel nomogram for predicting an aggressive histological type
Source: Front Oncol. 2025 Mar 14;15:1472250. doi: 10.3389/fonc.2025.1472250 (PMC11949794; doi:10.3389/fonc.2025.1472250)
Supplement: Supplementary file 1 [file Table1.docx]

**Supplementary table**

| **Table 1**│MRI protocol: sequences and parameters of institution 1. | | | | | |
| --- | --- | --- | --- | --- | --- |
| Parameters | T1WI | T2WI | FS-T2WI | DWI | Contrast-enhanced MRI |
| Repetition / echo time (msec) | 550/10 | 4000/83 | 8000/83 | 2800/81 | 4.89/2.38 |
| Sequence | TSE | TSE | TSE | EP2D | VIBE |
| Bandwidth (Hz) | 178 | 260 | 260 | 1250 | 400 |
| Thickness(mm) | 4 | 4 | 4 | 5 | 3 |
| Gap(mm) | 1.2 | 1.2 | 1.2 | 1.5 | 0.9 |
| Field of view (mm) | 350 | 350 | 350 | 300 | 380 |
| Flip angle (degrees) | 150 | 144 | 150 |  | 10 |

| **Table 2**│MRI protocol: sequences and parameters of institution 2. | | | | | |
| --- | --- | --- | --- | --- | --- |
| Parameters | T1WI | T2WI | FS-T2WI | DWI | Contrast-enhanced MRI |
| Repetition / echo time (msec) | 400/18 | 4550/107 | 4019/100 | 2919.2/66.3 | 4.0/1.5 |
| Sequence | TSE | TSE | TSE | EPI | mDIXON |
| Bandwidth (Hz) | 291 | 1161.2 | 918.5 | 52.8 | 1430.8 |
| Thickness(mm) | 6 | 4 | 4 | 5 | 3 |
| Gap(mm) | 1.5 | 0.5 | 1.5 | 1.5 | -2.5 |
| Field of view (mm) | 240 | 280 | 250 | 240 | 250 |
| Flip angle (degrees) | 90 | 90 | 90 | 11.2 | 10 |
